# Progestin and Nuclear Progestin Receptor Are Essential for Upregulation of Metalloproteinase in Zebrafish Preovulatory Follicles

**DOI:** 10.3389/fendo.2018.00517

**Published:** 2018-09-18

**Authors:** Dong Teng Liu, Nichole J. Carter, Xin Jun Wu, Wan Shu Hong, Shi Xi Chen, Yong Zhu

**Affiliations:** ^1^State Key Laboratory of Marine Environmental Science, College of Ocean and Earth Sciences, Xiamen University, Xiamen, China; ^2^Department of Biology, East Carolina University, Greenville, NC, United States

**Keywords:** ovulation, *adamts9*, progestin, Pgr, metalloproteinase

## Abstract

Ovulation requires proteinases to promote the rupture of ovarian follicles. However, the identity of these proteinases remains unclear. In our previous studies using RNA-seq analysis of differential expressed genes, we found significant down-regulation of five metalloproteinases: *adam8b* (a disintegrin and metalloproteinase domain 8b), *adamts8a* (a disintegrin and metalloproteinase with thrombospondin motif 8a), *adamts9, mmp2* (matrix metalloproteinase 2), and *mmp9* in the nuclear progestin receptor knockout (*pgr*^−/−^) zebrafish that have failed to ovulate. We hypothesize that these metalloproteinases are responsible for ovulation and are regulated by progestin and Pgr. In this study, we first determined the expression of these five metalloproteinases and *adamts1* in preovulatory follicles at different times within the spawning cycle in *pgr*^−/−^ and wildtype (*wt*) zebrafish and under varying hormonal treatments. We found that transcripts of *adam8b, adamts1, adamts9*, and *mmp9* increased drastically in the preovulatory follicular cells of *wt* female zebrafish, while changes of *adamts8a* and *mmp2* were not significant. This increase of *adam8b, adamts9*, and *mmp9* was significantly reduced in *pgr*^−/−^, whereas expression of *adamts1* was not affected in *pgr*^−/−^ zebrafish. Among upregulated metalloproteinases, *adamts9* mRNA was found to be expressed specifically in follicular cells. Strong immunostaining of Adamts9 protein was observed in the follicular cells of *wt* fish, and this expression was reduced drastically in *pgr*^−/−^. Interestingly, about an hour prior to the increase of metalloproteinases in *wt* fish, both Pgr transcript and protein increased transiently in preovulatory follicular cells. The results from *in vitro* experiments showed that *adamts9* expression markedly increased in a dose, time and Pgr-dependent manner when preovulatory follicles were exposed to a progestin, 17α,20β-dihydroxy-4-pregnen-3-one (DHP). Taken together, our results provide the first evidence that upregulation of *adamts9* occurs specifically in preovulatory follicular cells of zebrafish prior to ovulation. Progestin and its receptor (Pgr) are essential for the upregulation of metalloproteinases.

## Introduction

Ovulation is essential for successful reproduction to occur. In vertebrates, ovulation is triggered by a surge of luteinizing hormone (LH) and mediated by progesterone (P4) (1–5). Ovulation also requires upregulation of proteolytic enzymes (6). These proteinases promote the rupture of follicles releasing mature oocytes. Studies have shown correlations between increased expression of proteolytic enzymes and elevated levels of P4 or its receptor (a.k.a. the progestin receptor, PGR). For instance, induction of transcripts of *Adamts1* (a disintegrin and metalloproteinase with thrombospondin motifs 1) by human chorionic gonadotropin (hCG), a popular substitute for LH, was reduced in PGR knockout (*Pgr*^−/−^) mice (7). A synthetic PGR agonist, R5020, could reverse the inhibitory effect of mifepristone (RU486) on the LH induced expression of *ADAMTS1* and *ADAMTS9* mRNA in granulosa cells of cattle (8). In a teleost medaka, *mmp15* (matrix metalloproteinase 15) was found to be upregulated by Pgr within LH exposed follicles (9). Further, plasminogen activator and several other MMPs were also reported to be regulated by PGR during ovulation (3, 10, 11). Though the relationships between PGR and these proteases have implicated their involvement in the ovulation process, our knowledge of the regulation and functions of these proteases is extremely limited. So far, knockout studies of these metalloproteinases in mice have provided little information on the functions of these proteases, mainly due to null mice either dying *in utero* or exhibiting no observable defects (12–16). One exception is ADAMTS1 knockout mice that were found to be sub-fertile, but ultimately they were still able to ovulate (17). By comparison, anovulatory PGR knockout mice were completely infertile (18). Furthermore, ADAMTS1 is not expressed in cumulus cells (19), ADAMTS1 gene does not have P4 receptor response element (20) and is believed not to be regulated by Pgr in high mammalian species (personal communication). These studies suggest that there may be critical protease(s) other than ADAMTS1 necessary for ovulation that has not yet been identified.

Zebrafish is an established model for studying gene functions and signaling pathways in conserved ovarian events such as oogenesis, oocyte maturation, and ovulation (21, 22, 23). Our previous results show that *pgr*^−/−^ female zebrafish were unable to ovulate demonstrating the conserved function of Pgr in ovulation from fish to mice (18, 24, 25). Furthermore, compared to *pgr*^−/−^ zebrafish, a genome-wide analysis of transcripts revealed conserved signaling pathways and higher expression levels for *adamts8a, adamts9, mmp2, mmp9*, and *adam8b* (a disintegrin and metalloproteinase domain 8b) in the follicular cells of wildtype (*wt*) fish (26). We hypothesized that some of these genes are likely obligatory for follicular rupture and ovulation in zebrafish. In this study, we aimed to elucidate the expression and hormonal regulation of these proteinases in zebrafish ovarian follicles. We first determined the expression changes of five abovementioned metalloproteinases and *adamts1*. We found dramatic increases of *adam8b, adamts1, adamts9*, and *mmp9* in the follicular cells prior to ovulation. Interestingly, expression of *adamts9, adam8b*, and *mmp9* were significantly reduced in *pgr*^−/−^ fish, whereas that of *adamts1* was not affected. Then, we found that 17α,20β-dihydroxy-4-pregnen-3-one (DHP) stimulated *adamts9* expression in preovulatory follicles in a dose, time and Pgr-dependent manner. Our study suggests progestin and Pgr are critical for the upregulation of metalloproteinases prior to ovulation in zebrafish.

## Materials and methods

### Zebrafish husbandry

The *wt* zebrafish used in this study are a Tübingen strain initially obtained from the Zebrafish International Resource Center and propagated in our lab. Pgr gene knockout lines used in this study were generated and characterized previously (24, 27). Fish were kept under a photoperiod of 14 h (h) light and 10 h dark (lights on at 09:00, lights off at 23:00), at a water temperature around 28.5°C, pH ~7.2, and salinity conductivity ranging from 500 to 1,200 μS in automatically controlled zebrafish rearing systems (Aquatic Habitats Z-Hab Duo systems, Florida, USA). Fish were fed three times daily to satiation with a commercial food (Otohime B2, Reed Mariculture, CA, USA) containing high protein content and supplemented with newly hatched brine shrimp *Artemia* (Brine Shrimp Direct, Utah, USA). The Institutional Animal Care and Use Committees (IACUC) at both Xiamen University and East Carolina University have approved all experimental protocols.

### Collection of stage I–IV follicles

Thirty mature females from *wt* or *pgr*^−/−^ were collected at 08:00 (1 h before lights turned on) from group housed tanks in conditions described as in section Zebrafish Husbandry. These fish were deeply anesthetized in a lethal dose of MS-222 (300 mg/L buffered solution) for 10 min. To ensure death, the spinal cord and blood supply behind the gill cover were cut off using sharp scissors. Ovaries were removed immediately and placed in a 90-mm petri dish containing 60% L-15 media (Sigma, in 15 mM HEPES, pH 7.2). Thereafter, ovaries were cut into ~4 mm^3^ pieces, and then transferred to a 15-ml centrifuge tube. Individual oocytes were separated by pipetting up and down using a disposable glass pipette with a polished 3 mm opening. Hereafter, we will use “follicles” to specifically refer to follicular cells and their enclosed oocytes to distinguish them from follicular cells or defolliculated oocytes (i.e., denuded oocytes). Individual follicles of various sizes (Table 1) were separated into five stages according to an established oocyte classification system in zebrafish with modification (28). We further subdivided stage IV follicles into two stages, immature stage IVa (before germinal vesicle breakdown, i.e., GVBD) and mature stage IVb (after GVBD has occurred but prior to ovulation). Though the outside appearance of stage IVb follicles is transparent, same as stage V, distinguishing stage IVb follicles from stage V mature ova is straightforward since stage IVb mature follicles are scattered around the ovary, surrounded by follicular cells and other immature follicles. Whereas, stage V ovulated oocytes are grouped together and pushed to the posterior part of the ovary *in vivo* (26). Stage V ovulated oocytes were not collected for two reasons: (1) Our targeted genes are expressed mainly in the follicular cells, and stage V mature oocytes do not have follicular cells; (2) It was not possible to collect stage V oocytes from *pgr*^−/−^ fish due to their inability to ovulate.

**Table 1:** Classification of different developmental stages of zebrafish follicles.

**Developmental stage^a^**	**Size range (μm)**	**Characteristic**	**Size selected (μm)**
I	20~140	Primary growth	<140
II	140~340	Previtellogenic	~250
III	340~650	Vitellogenic	400~450
IV a	650~720	Fully grown but immature, yolk opaque, GV visible^b^	>650
IV b	650~720	Mature, yolk translucent, GVBD occurred^c^	>650
V	720–750	Ovulated egg	–

a *Modified from Selman et al. (28)*.

b. GV, germinal vesicle;

c. *GVBD, germinal vesicle breakdown*.

### Collection of follicular cells and denuded oocytes from stage IV follicles during a spawning cycle

In group housing conditions, zebrafish skip spawning frequently in part due to intense competition for space and food. To increase and monitor individual spawning, we set up multiple spawning tanks with a pair of approximate 3-month old mature male and female *wt* fish for each tank. Everyday around 22:00 (1 h prior to lights off), water in spawning tanks was replaced with clean water along with an inner tank insert that allows fertilized eggs to drop through to the bottom to prevent the eating of the eggs by the adults. Spawning and release of fertilized eggs (~150 embryos/day) were visually confirmed and recorded for each pair of fish every morning. At 12:00, fish were transferred to a new spawning tank with clean water but without an insert, so they could access the commercial fish food and newly hatched brine shrimp. Fish food and brine shrimp were supplemented every 3 h. In this setup and enhanced feeding condition, the majority of the pairs (7–8 out of 10 pairs) spawn almost daily.

At 1 week following the setup of spawning, the mature female zebrafish of the pairs were sacrificed, their ovaries removed, and stage IV follicles were collected as described in section Zebrafish Husbandry. Stage IV follicles from *wt* fish were collected at four different time points: 13:00 (stage IVa, from fish that skipped spawning that morning); 21:00 (stage IVa, 2 h before lights off); 06:00 (stage IVa, onset of oocyte maturation and 3 h before lights on); and 08:00 (stage IVb, after oocytes have matured but before ovulation, 1 h before lights on). To determine the effect of Pgr knockout, stage IVb follicles at 08:00 were also collected from 3-month old virgin female *pgr*^−/−^ fish that were paired with fertile *wt* male at the same time. Follicular cells (collected from ~100 follicles/fish) and their enclosed oocytes (collected from ~10 follicles/fish) were separated from stage IV follicles using a pair of small glass needles. Respective samples were pooled and homogenized immediately in RNAzol solution according to an established procedure (26, 29).

### Various hormone treatments of stage IVa fully-grown immature follicles *in vitro*

Stage IVa follicles (>650 μm) with visible germinal vesicles (GV) were collected from females at ~05:30. Briefly, intact follicles with no obvious damage were selected and transferred into a 24-well tissue culture plate containing 60% L15 medium (25 follicles per well). These follicles were incubated for 2.5 h at 25°C with DHP (1–1,000 nM), testosterone (T, 500 nM), or RU486 (0.01–10 μM), alone or in combination. An exposure time of 2.5-h was selected to conduct the various hormone treatment based on results from our time course experiments (up to 6 h). Follicles that underwent final oocyte maturation, indicated by transparent yolk and GVBD, were easily determined under a dissecting microscope at the end of incubation. Excluding broken follicles, the number of transparent follicles that completed GVBD were counted and presented as a percentage of the total follicles. Thereafter, all the follicles were collected and homogenized immediately in RNAzol for qPCR analyses of gene expression, or in 1X SDS sample buffer for Western blot analyses of protein expression.

### RNA extraction, reverse transcription, and real-time quantitative PCR

Total RNA was extracted using RNAzol (MRC, Cincinnati, Ohio, USA) and a Qiagen RNeasy kit. According to the manufacturer's protocol, BAN solution (4-bromoanisole) was added to purify the RNA and eliminate genomic DNA following the first precipitation step with water. After the second precipitation, an equal volume of cold 100% ethanol was added. The mixture was then loaded onto a RNeasy free spin column, centrifuged (8,000 g, for 30 s), washed twice with 650 μL 75% ethanol, and eluted in RNase-free water. We used 15–30 μL depending on the initial amount of sample. The approximate concentration and purity of samples were determined using a Nanodrop 2000 Spectrophotometer. RNA samples with concentrations >100 ng/μL, OD 260/280 >1.8, and OD 260/230 >1.3 were retained for further analyses. Reverse transcription was performed using SuperScript III Reverse Transcriptase and 0.5 μg of total RNA from each sample in a reaction volume of 10 μL, per manufacturer's instructions (Invitrogen, Carlsbad, CA). Gene expression was determined by quantitative real time PCR (qPCR) analyses according to previously established protocols (26, 29) using gene specific qPCR primers for targeting genes for *adam8b, adamts1, adamts8a, adamts9, mmp2, mmp9*, and *pgr* (Table 2). To avoid genomic DNA contamination, forward and revise primers were designed to be in two different exons of each target gene. Authentic single qPCR product for each gene was confirmed by melting curve analyses, gel electrophoresis, and sequencing. The absolute transcript levels, expressed as copies/μg total RNA, were determined using Ct-values of samples and standard curves corresponding to different target genes generated from known serial plasmid concentrations (10^2^-10^7^ copies/μL). We did not use the comparative Ct method for this study because house-keeping-genes including *actb1, actb2*, and *ef1a* vary between different developmental stages of follicles (data not shown).

**Table 2:** Primers used in the study.

**Gene symbol**	**Accession number**	**Forward primer (5^′^⟶3^′^)**	**Reverse primer (5^′^⟶3^′^)**
*adam8b*	XM_003199573	CCTGGCATCCACAATTGCAC	CATTACCACAGACAGGCCCA
*adamts1*	XM_688443	ACACCGTGCACTCCAGATTC	GGCTACGGCCTCCAAAAGAT
*adamts8a*	XM_021476490	ACTCTTCCCTGGTCTCCATGT	CAGCAGCTAAAGGGATGGTCA
*adamts9*	NM_001257196	TCACCCAACCCCGATTTTCG	CAAGAGCGCTGTTCAATGGG
*mmp2*	NM_198067	CCCGATGACCTAGATGGTGC	TTTGACCTCGCCGACTTTGA
*mmp9*	NM_213123	TCTGCCTTTGAGGACCACCT	CCGAAAGCTGCATCAGTGAA
*pgr*	NM_001166335	ACAGACAGCATACACCGC	TCCACAGGTCAGAACTCC

### Western blotting

Total protein was extracted from 10 stage IVa or IVb follicles from newly sacrificed fish at various time points during an ovulatory cycle, or from *in vitro* incubation. Collected follicles were homogenized immediately by sonication in 100 μl of 1X SDS sample buffer (62.5 mM Tris-Cl pH 6.8, 2% SDS, 10% glycerol, 100 mM Dithiothreitol) with 0.1% protease inhibitor cocktail (Sigma, Saint Louis, MO) on ice for about 10 short bursts (1–2 s for each burst, Sonic Dismembrator, Fisher Scientific). Samples were then boiled immediately for 10 min and stored in −20°C until analysis. Samples were loaded onto an 8% SDS PAGE gel (10 μL is equivalent to one follicle), separated by electrophoresis, and transferred to a nitrocellulose membrane. The membrane was pre-incubated for 2 h with a blocking solution containing 5% BSA (albumin from bovine serum, Sigma-Aldrich Catalog# A7906) in TBST (50 mM Tris, 100 mM NaCl, 0.1% Tween 20, pH 7.4). Then incubated overnight with a primary antibody [zebrafish PGR primary antibody, 1:250 dilution (29); or α-tubulin primary antibody, Sigma catalog#T5168, 1:5,000 dilution] in 1% BSA blocking solution. The following day, the membrane was washed with 1X TBST five times for 5 min each, incubated for 1 h with horseradish peroxidase (HRP) conjugated secondary antibody (goat anti-mouse antibody HRP for α-tubulin, Fisher catalog# PI31430, 1:5,000 dilution; goat anti-rabbit antibody HRP for Pgr, Cell Signaling catalog#7074S, 1:3,000 dilution). Then washed again with TBST buffer five times for 5 min each. The membrane was developed using Super Signal West Extended Dura Substrate (Pierce, Rockford, IL) and visualized using a Fluor Chem 8900 imaging station (Alpha Innotech, San Leandro, CA). Protein size was calibrated using a biotinylated protein ladder (Cell Signaling) and a pre-stained protein ladder (Fermentas).

### Immunohistochemistry and hematoxylin and eosin (HE) stain

Two commercial antibodies for human ADAMTS9 were used in the immunohistochemical staining (Triple Point Biologics, Forest Grove, Oregon, USA). These antibodies target domains in human ADAMTS9 that share sequence homology to those in zebrafish *adamts9*. One antibody, catalog# RP5-ADAMTS-9, targets 44 amino acid residues of metalloprotein domain, and shares 85.7% sequence homology with zebrafish Adamts9. The other antibody, catalog# RP1-ADAMTS-9, targets the 44 amino acid residues of propeptide domain and shares 41% sequence homology with zebrafish Adamts9. Mature ovaries for the studies were collected from *wt* or *pgr*^−/−^ fish at 08:00 (1 h prior to lights on) and fixed in 10% buffered formalin for overnight. Samples were washed in tap water for 30 min, dehydrated through increasing concentrations of ethanol, and embedded in paraffin. A series of 8-μm sections were made deparaffined in xylene, rehydrated in decreased concentration of ethanol, then subjected to HE stains (hematoxylin staining for 2 min, rinsed in running tap water for 5 min, and eosin staining for 30 s), or immunohistological staining according to previously established protocols with a few modifications (24, 30). Briefly, sections were deparaffinized sequentially by 5-min treatment with 100% xylene (twice), 100% ethanol (twice), 95% ethanol, 70% ethanol, and PBS (137 mM NaCl, 2.7 mM KCl, 10 mM Na_2_HPO_4_·12H_2_O, 2 mM KH_2_PO_4_) twice for each section. Then sections were incubated with 0.1% testicular hyaluronidase (Sigma catalog# H3506, 1 mg/mL in 30 mM sodium acetate, 125 mM sodium chloride, pH 5.2) at 37°C for 1 h for antigen retrieval. Thereafter, sections were washed with PBS twice, then incubated in 0.3% H_2_O_2_ for 30 min to reduce endogenous activities of horseradish peroxidase. Following three washes with PBS, sections were blocked with normal goat serum for 30 min, and then incubated with ADAMTS9 primary antibodies (1:1,000 dilution for RP5-ADAMTS-9; 1:250 dilution for RP1-ADAMTS-9) or normal rabbit serum (1:1,000 dilution) overnight. After three washes with PBS, immunoreaction was developed using the Vectastain ABC kit (Vector Laboratories, Burlingame, California) according to the manufacturer's protocol.

### Statistical analyses

For the comparison of two data sets (e.g., *wt* vs. *pgr*^−/−^, or DHP vs. vehicle control), unpaired students' *t*-test was used to determine significant differences. For multiple group comparison, one-way ANOVA followed by Tukey's test or Dunnett's test was used. All experiments were repeated at least three times, and the results of one representative experiment is shown in the following section.

## Results

### Changes of metalloproteinase expression in stage I–IV follicles in mature female zebrafish

**Figure 1:**
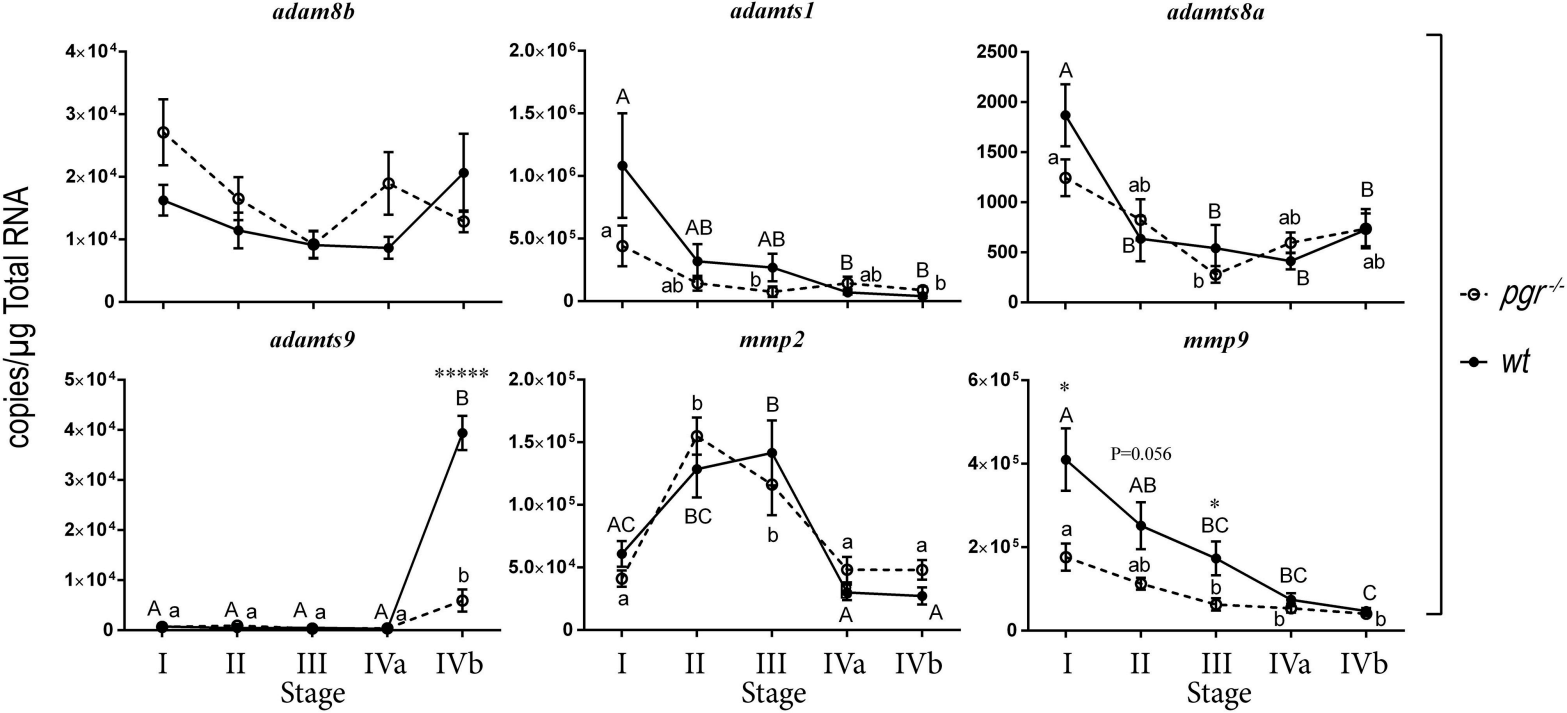
Expression of representative metalloproteinases in various stages of follicles (follicular cells-enclosed oocytes) in wildtype (*wt*, solid line with filled circles) and Pgr knockout zebrafish (*pgr*^−/−^, dashed line with open circles) *in vivo*. All transcripts were determined and expressed as absolute value according to standard curves established with known concentrations of serially diluted plasmids using real-time quantitative PCR (qPCR). Stages of zebrafish follicles were classified per Selman et al. (28). Stage IV follicles were further sub-divided into stage IVa and stage IVb. Stage IVa follicles were fully-grown immature follicles prior to oocyte maturation. Stage IVb follicles were follicular cells enclosed mature oocytes that have gone through oocyte maturation, but not yet ovulated (also see Table 1 for detail). Different letters (uppercase for *wt*; lowercase for *pgr*^−/−^) indicate significant differences among different follicular stages in *wt* or *pgr*^−/−^ fish. Significant differences between *wt* or *pgr*^−/−^ fish at the same developmental stage of follicles are indicated by asterisks. *p < 0.05, *****p < 0.00001. *N* = 6. The metalloproteinases examined were *adam8b*, a distintegrin and metalloproteinase domain 8b; *adamts1*, a disintegrin and metalloproteinase with thrombospondin type 1 motif 1; *adamts8a*; *adamts9*; *mmp2*, matrix metalloproteinase 2; and *mmp9*, matrix metalloproteinase 9, respectively.

We hypothesized that metalloproteinases required for ovulation should be expressed highly in late stage follicles such as stage IVa or IVb follicles. Therefore, we separated follicles according to their sizes and stages, and determined expression of six representative metalloproteinases in early (stage I) through late stage (IVb) follicles (Figure 1). Expression of *adamts9* remained at extremely low levels (<300 copies/μg total RNA) and was sometimes undetectable from stage I through stage IVa fully-grown immature follicles; however, its expression increased drastically in stage IVb mature follicles in both *wt* and *pgr*^−/−^ fish. This increased expression of *adamts9* in *pgr*^−/−^ (3,000–4,000 copies/μg total RNA) was only one-tenth of that in *wt* fish (~40,000 copies/μg total RNA). Intriguingly, high expression of *adam8b, adamts1, mmp2*, and *mmp9* were observed in all stages of follicles (>10,000 copies/μg total RNA), whereas expression of *adamts8a* remained at low levels (<2,000 copies/μg total RNA). Surprisingly, none of these five metalloproteinases (*adam8b, adamts1, adamts8a, mmp2, mmp9*) showed significant difference in their expression between *pgr*^−/−^ and *wt*.

### Pgr mediates the upregulation of metalloproteinases in preovulatory follicular cells

**Figure 2:**
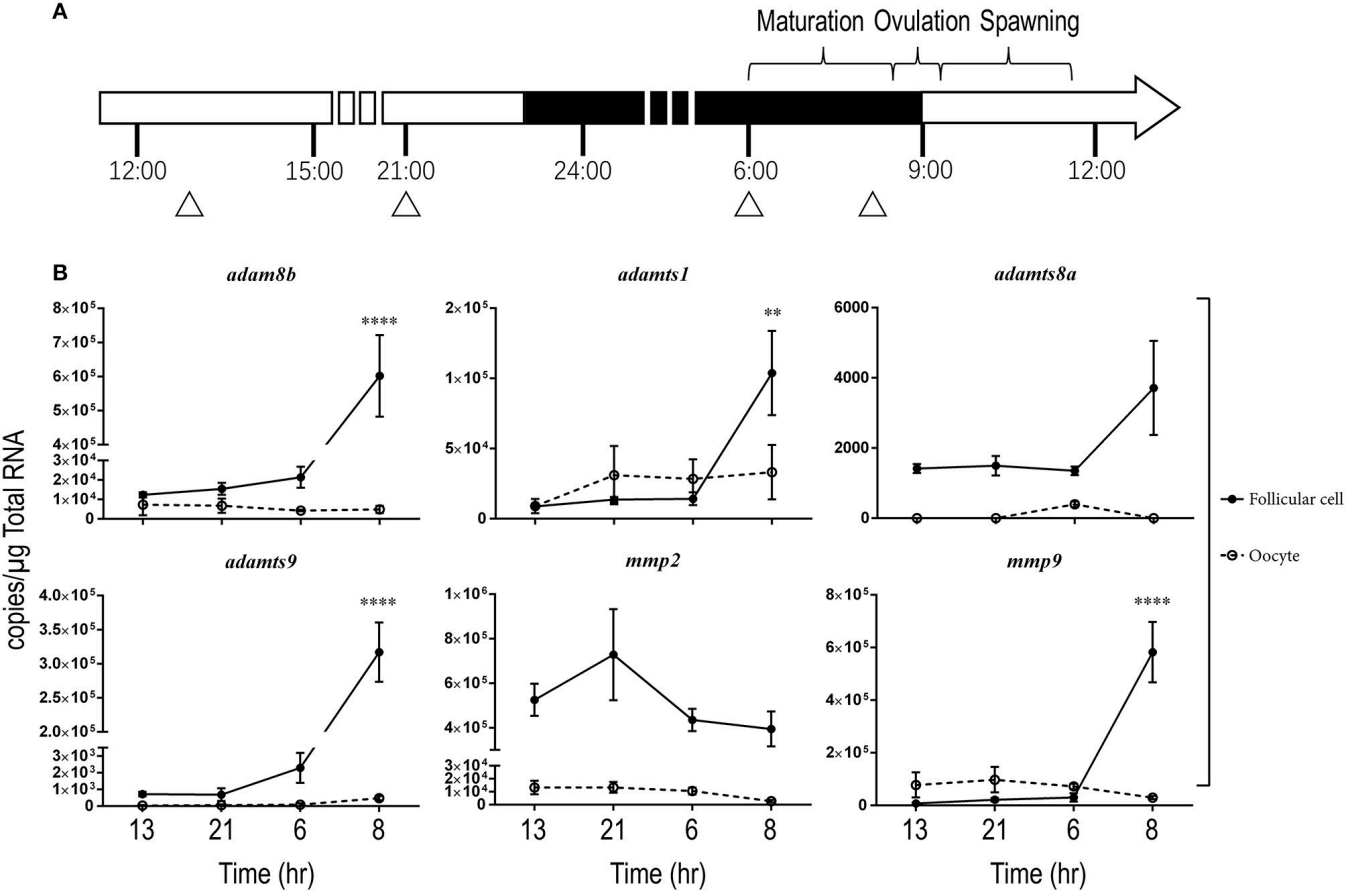
Expressions of *adam8b, adamts1, adamts9*, and *mmp9* transcripts significantly increased in stage IV preovulatory follicular cells during a daily spawning cycle in wildtype (*wt*) fish *in vivo*. Transcripts of representative metalloproteinases were determined in stage IV follicular cells (solid line with filled circles) or denuded oocytes (dash line with open circles) separately during a daily spawning cycle in *wt* fish. **(A)** A schematic drawing showing the daily ovulatory cycle of zebrafish. Day and night cycles are indicated by open or black bars, respectively. Lights were switched on at 09:00 and off at 23:00. Approximate time for daily oocyte maturation, ovulation and spawning in zebrafish are indicated on top of the bar. Samples were collected from four representative time points, indicated by triangles: 13:00 (after spawning), 21:00 (2 h before lights off), 06:00 (prior to oocyte maturation), and 08:00 (after oocyte maturation but prior to ovulation). **(B)** Expression of representative metalloproteinase transcripts were determined by qPCR. Asterisks indicate a significant increase of transcripts compared to the previous sampling time point in follicular cells (solid line with black circles). No significant change was observed in the denuded oocytes (dash line with open circles). **p < 0.01, ****p < 0.0001. *N* = 5.

We then hypothesized that changes of metalloproteinases in late stage follicles, especially in the follicular cells, could be important for ovulation but their activity may be masked by differential expression and/or high levels of metalloproteinase transcripts in large oocytes when entire follicles (follicular cells and their enclosed oocytes) are used. Hence, we focused on changes of transcripts in stage IVa and IVb fully grown follicles. We also separated the follicular cell layers from their enclosed oocytes and determined gene expression in both cell types during the ovulatory cycle in *wt* female zebrafish (Figure 2). In follicular cells, expression of *adam8b, adamts1, adamts9*, and *mmp9* remained relatively low at most times but increased significantly at about 1 h (08:00) prior to ovulation that occurred at around 09:00 when lights were turned on. The changes of *adamts8a* and *mmp2* transcripts were not significant during the daily spawning cycle. In denuded oocytes, the expression of *adamts8a* and *adamts9* was extremely low and nearly undetectable (<300 copies/μg total RNA). By contrast, expression of *adam8b, adamts1, mmp2*, and *mmp9* in denuded oocytes was relatively high (>5,000 copies/μg total RNA) but did not significantly change during the ovulatory cycle.

**Figure 3:**
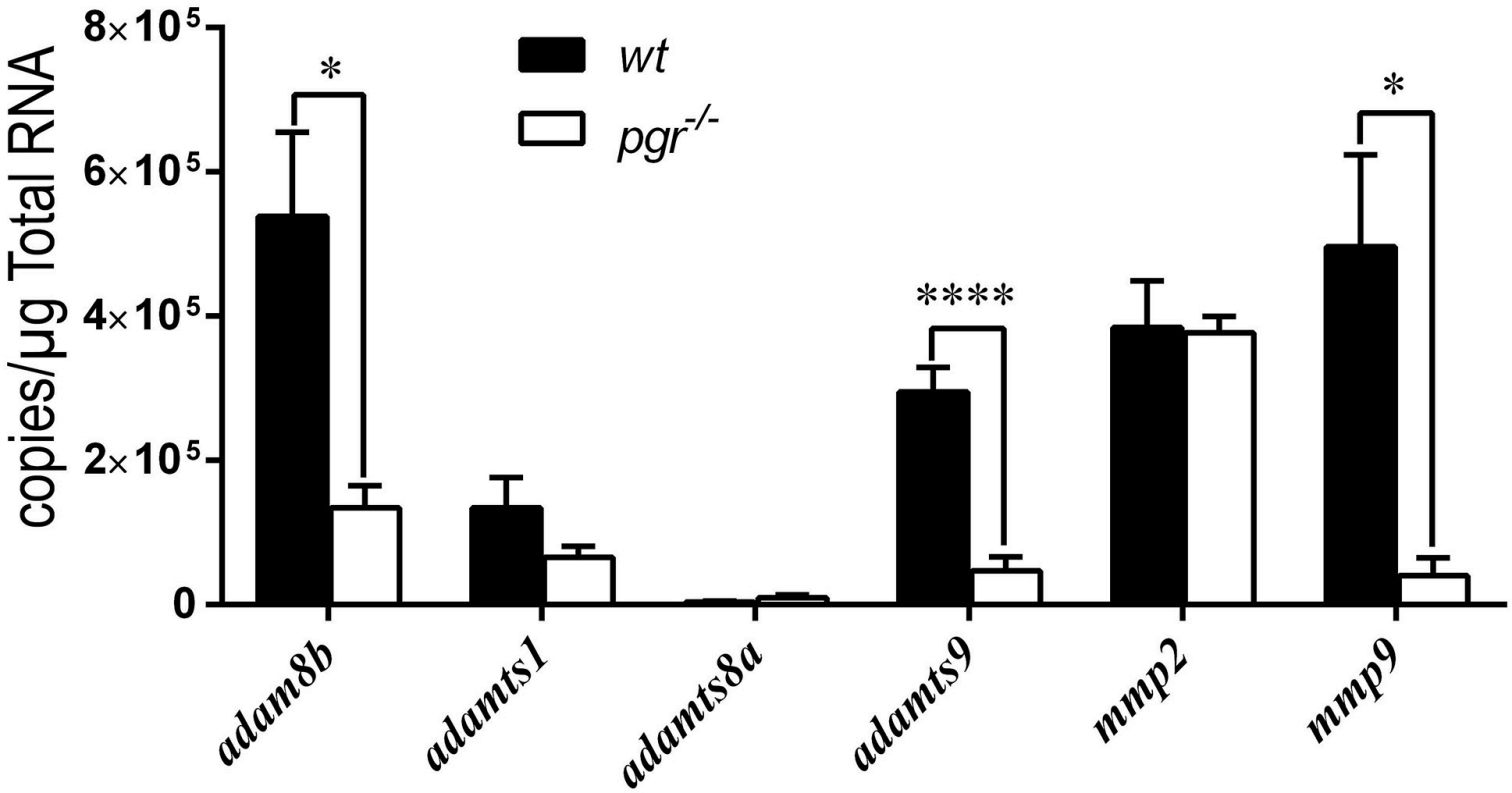
Expressions of *adam8b, adamts9*, and *mmp9* were significantly reduced in the follicular cells of preovulatory follicles (stage IVb) in *pgr*^−/−^ than those in wildtype (*wt*) *in vivo*. Transcripts of representative metalloproteinases were determined by quantitative real-time PCR (qPCR) in follicular cells of stage IVb preovulatory follicles (at 08:00) in both *wt* and *pgr*^−/−^. Asterisks indicate significant differences between follicular cells of *wt* and *pgr*^−/−^. *p < 0.05, ****p < 0.0001. *N* = 6.

Because the expression of metalloproteinases was upregulated in preovulatory stage IVb follicles (mature but not ovulated follicles) sampled at 08:00, we hypothesized that Pgr is an upstream regulator for these metalloproteinases. We found significantly reduced expression of *adam8b, adamts9*, and *mmp9* in follicular cells of *pgr*^−/−^ fish compared to those in *wt* fish (Figure 3). Expression of *adamts1* was not affected by Pgr knockout as ADAMTS1 is not regulated by PGR in high mammalian species. In denuded oocytes, the expression of all aforementioned metalloproteinases was not significantly different between *wt* and *pgr*^−/−^ fish (data not shown).

### Changes of pgr expression during ovulatory cycle

**Figure 4:**
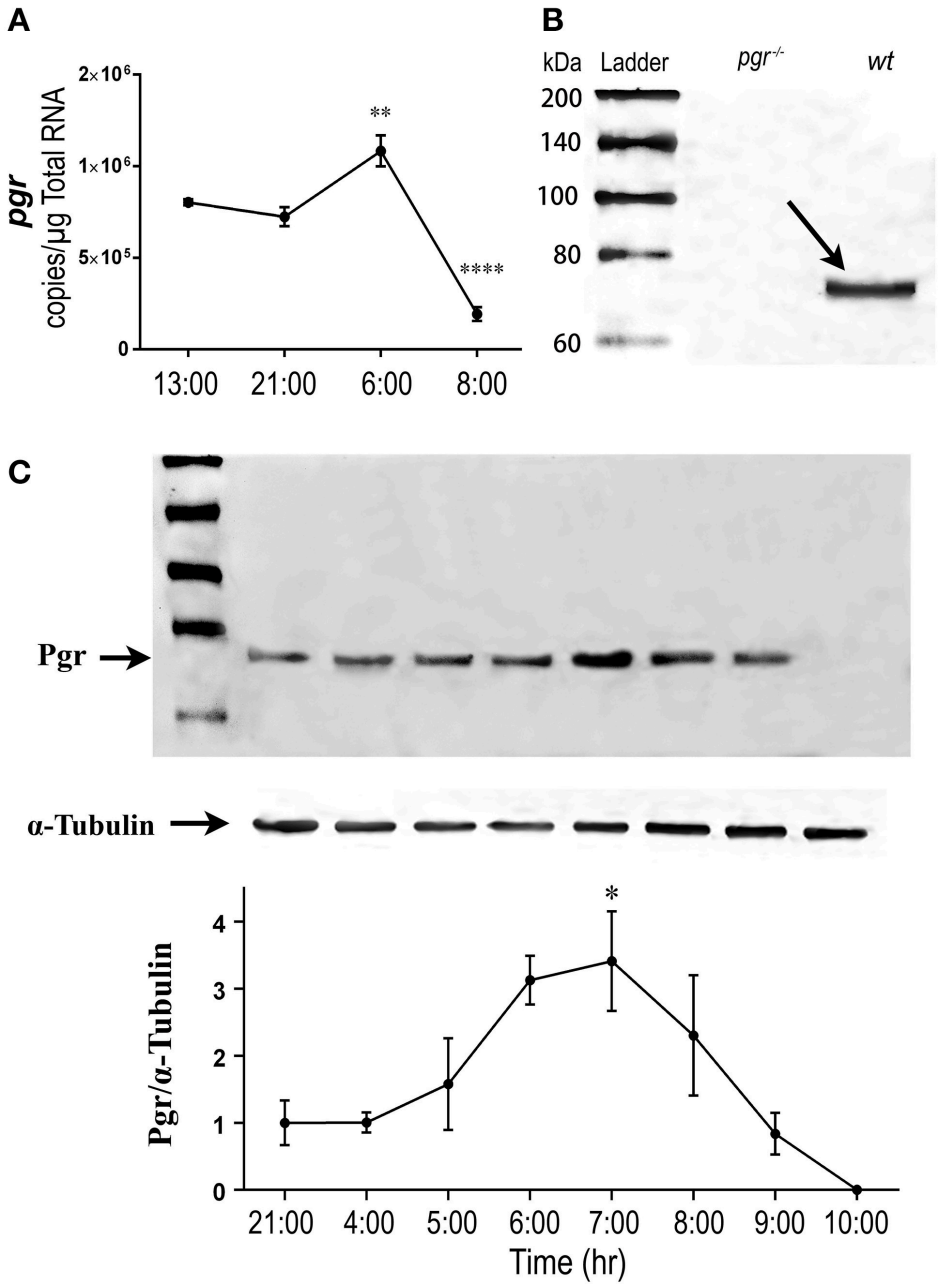
Transient increase of nuclear progestin receptor (Pgr) in follicular cells prior to ovulation *in vivo*. **(A)** Changes of *pgr* transcript. Transcripts were determined by quantitative real-time PCR (qPCR) in the follicular cells of stage IV follicles in an ovulatory cycle in wildtype (*wt*) zebrafish. Asterisks indicate significant change of transcripts compared to the previous time point. **p < 0.01, ****p < 0.0001. *N* = 5. **(B)** Western analysis of Pgr protein expression in stage IV follicles in *pgr*^−/−^ or *wt*. Arrow indicates the specific band of zebrafish Pgr at the approximate size of 69 kDa in *wt* fish. No band was observed in *pgr*^−/−^. **(C) (Top)** Representative images of a Western blot of Pgr proteins from stage IV (IVa or IVb) follicles sampled at various time of a spawning cycle. α-tubulin was used as an internal control for normalization of expression of Pgr protein; **(Bottom)** normalized Pgr expression at various time compared to Pgr expression at 21:00. Asterisks indicate significant difference compared to the value at 21:00. Experiment was repeated three times with results of one representative experiment presented as mean ± SEM. *p < 0.05. *N* = 3.

We hypothesized that upstream regulators of these metalloproteinases, especially Pgr would be upregulated prior to the upregulation of these metalloproteinases. As we predicated, expression of Pgr (transcript and protein) in follicular cells began to increase in the early morning (05:00, prior to maturation), reached a peak level in the middle of maturation (06:00–07:00), then decreased gradually thereafter (Figure 4).

### Exogenous DHP exposure directly upregulates *adamts9* in follicles *in vitro*

**Figure 5:**
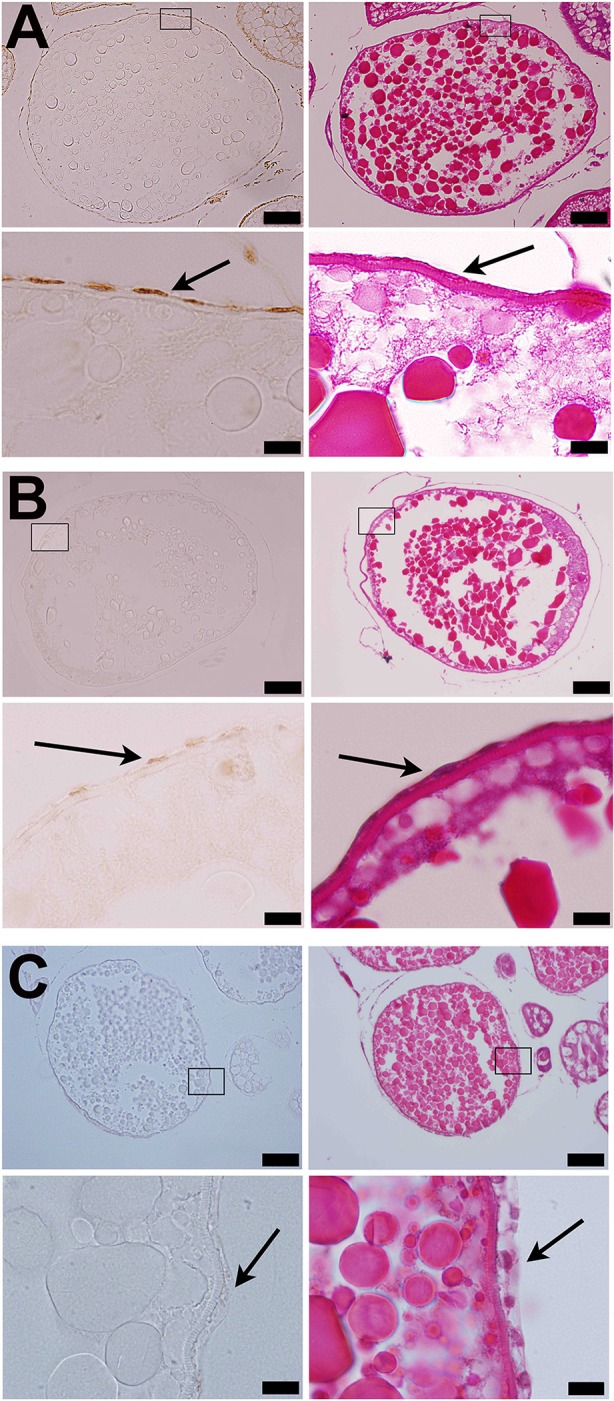
Strong immunostaining of Adamts9 was observed in the follicular cells of wildtype (*wt*) fish **(A)**. **(A)** Representative images of a stage IVb follicle stained with hematoxylin and eosin or immunostained with an antibody against metalloproteinase domain of human ADAMTS9 (RP5-ADMTS-9, Triple Point Biologics, Forest Grove, Oregon), **(B)** Representative images of a stage IVb follicle of Pgr-KO zebrafish fish stained at same time, **(C)** Negative control, representative images of a stage IVb follicle of wildtype zebrafish fish stained at same time but the primary antibody was replaced with normal rabbit serum. Pictures located on the bottom of panels are magnified images of the areas outlined on the top panels. Immunostaining are shown on the left side of panels, while HE stains of adjacent sections are shown on the right side of panels. Arrows indicate follicular cells. Similar results were obtained when RP1-ADAMTS-9 antibody used (data not shown).

To determine if low expression of metalloproteinases found in *pgr*^−/−^ zebrafish is due to direct effect of progestin and its receptor (Pgr) in the follicular cells, we chose an *in vitro* oocyte maturation assay (29), and focused on hormonal regulation of *adamts9* because only *adamts9* meets all following criteria: (1) Expressed specifically in follicular cells but not in the oocytes (Figures 2, 5); (2) Expressed differently between *wt* and *pgr*^−/−^ (Figures 2, 3, and 5); and (3) Increases significantly prior to ovulation (Figures 1, 2).

**Figure 6:**
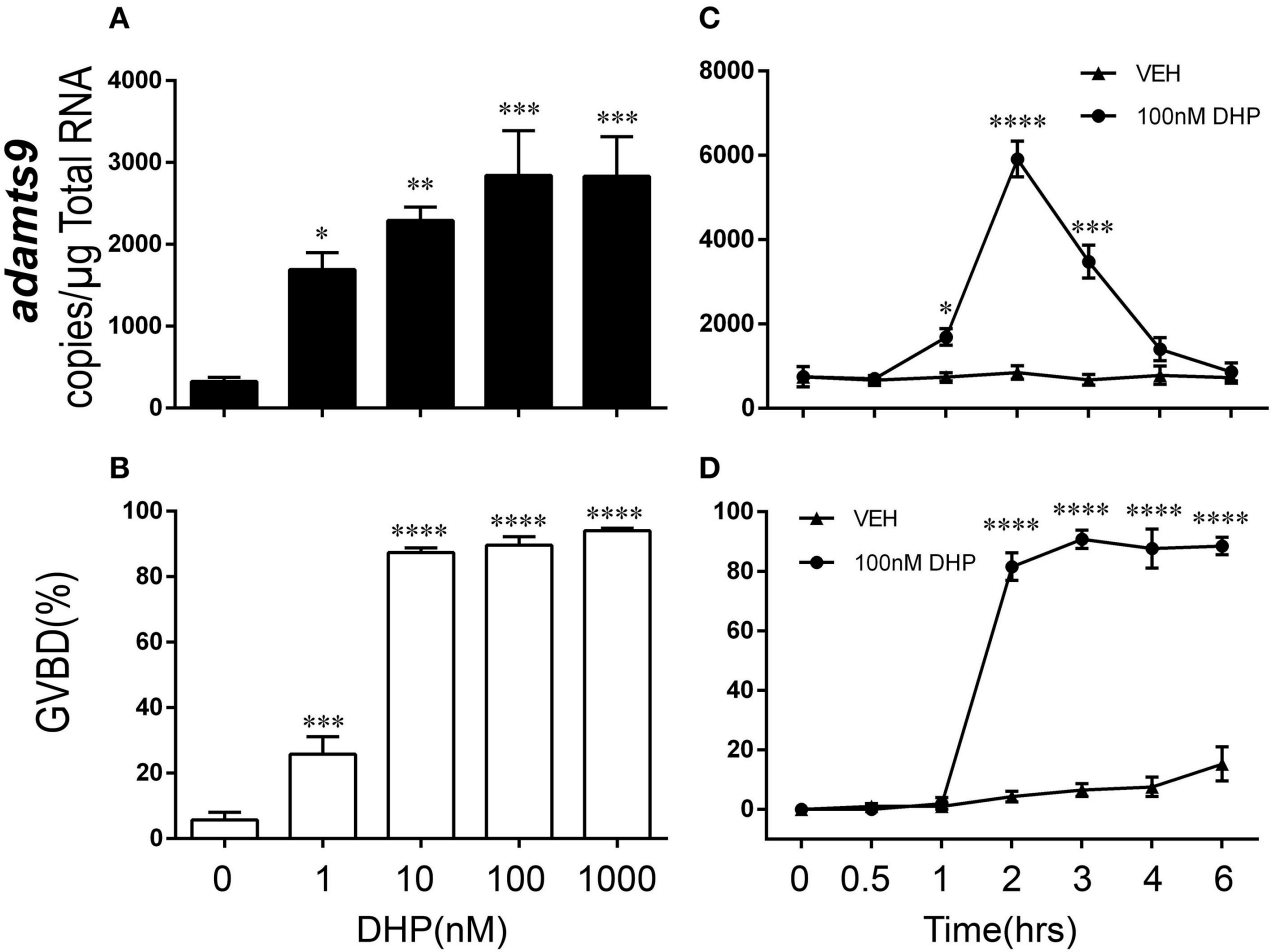
Dose- and time-dependent and transient increase of *adamts9* in stage IV follicles exposed to progestin in wildtype (*wt*) zebrafish *in vitro*. **(A,B)** Effects of various doses of progestin (17α,20β-dihydroxy-4-pregnen-3-one, DHP) on the expression of *adamts9* transcript and oocyte maturation, i.e., germinal vesicle breakdown (GVBD) during *in vitro* incubation. **(C,D)** Effects of DHP (100 nM) on *adamts9* expression and GVBD at various time points of *in vitro* incubation. Asterisks indicate significant difference in hormone treated samples compared to vehicle treatment at the same time point. *p < 0.05, **p < 0.01, ***p < 0.001, ****p < 0.0001. Experiment was repeated three times with results of one representative experiment presented as mean ± SEM. *N* = 4.

Consistent with expression of *adamts9* transcripts, expression of Adamts9 protein was also observed specifically in the follicular cells of zebrafish and was relative high in the follicular cells of *wt* than in *pgr*^−/−^ fish *in vivo* (Figure 5). Exposure of stage IVa follicles to DHP *in vitro* significantly upregulated *adamts9* expression in a dose dependent manner (Figure 6A). The increase of *adamts9* correlated well with the occurrence of oocyte maturation *in vitro* (Figure 6B). DHP-induced *adamts9* expression was transient (Figure 6C), peaking at 2 h post incubation with DHP (100 nM) when about 90% of stage IV oocytes had matured (Figure 6D), but then decreased gradually to basal levels after an additional 2 h of *in vitro* incubation.

### RU486 did not affect expression of *adamts9* but triggered oocyte maturation in follicles *in vitro*

**Figure 7:**
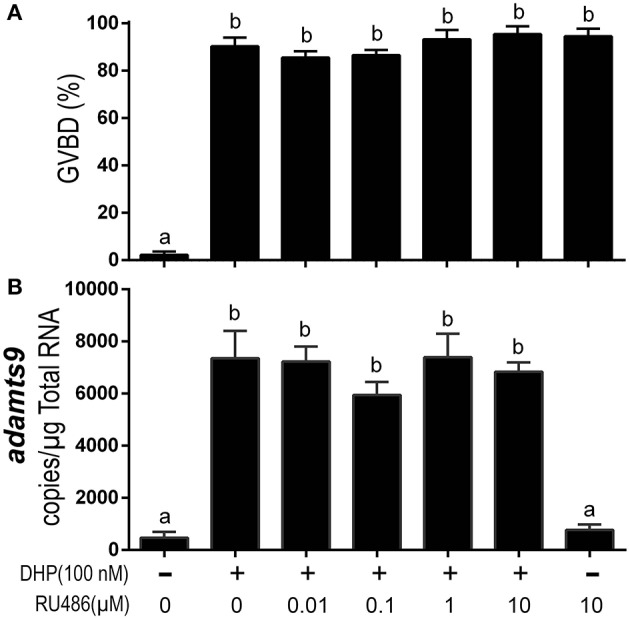
RU486 induced oocyte maturation but did not attenuate DHP-induced expression of *adamts9*. **(A)** Percentage of germinal vesicle breakdown (GVBD), **(B)** Expression of *adamts9* transcripts. Letters on the bars indicate significant differences (*p* < 0.0001). Experiment was repeated three times with results of one representative experiment presented as mean ± SEM. *N* = 4.

Exposure to the mammalian P4 antagonist, RU486, did not block DHP-induced oocyte maturation (Figure 7A). RU486 also did not inhibit *adamts9* expression induced by DHP (Figure 7B). Intriguingly, at a high dosage (10 μM) by itself, RU486 induced oocyte maturation, but had no effect on the expression of *adamts9* (Figure 7).

### Effect of DHP on the increased expression of *adamts9* is blocked in pgr*^−/−^* fish *in vitro*

**Figure 8:**
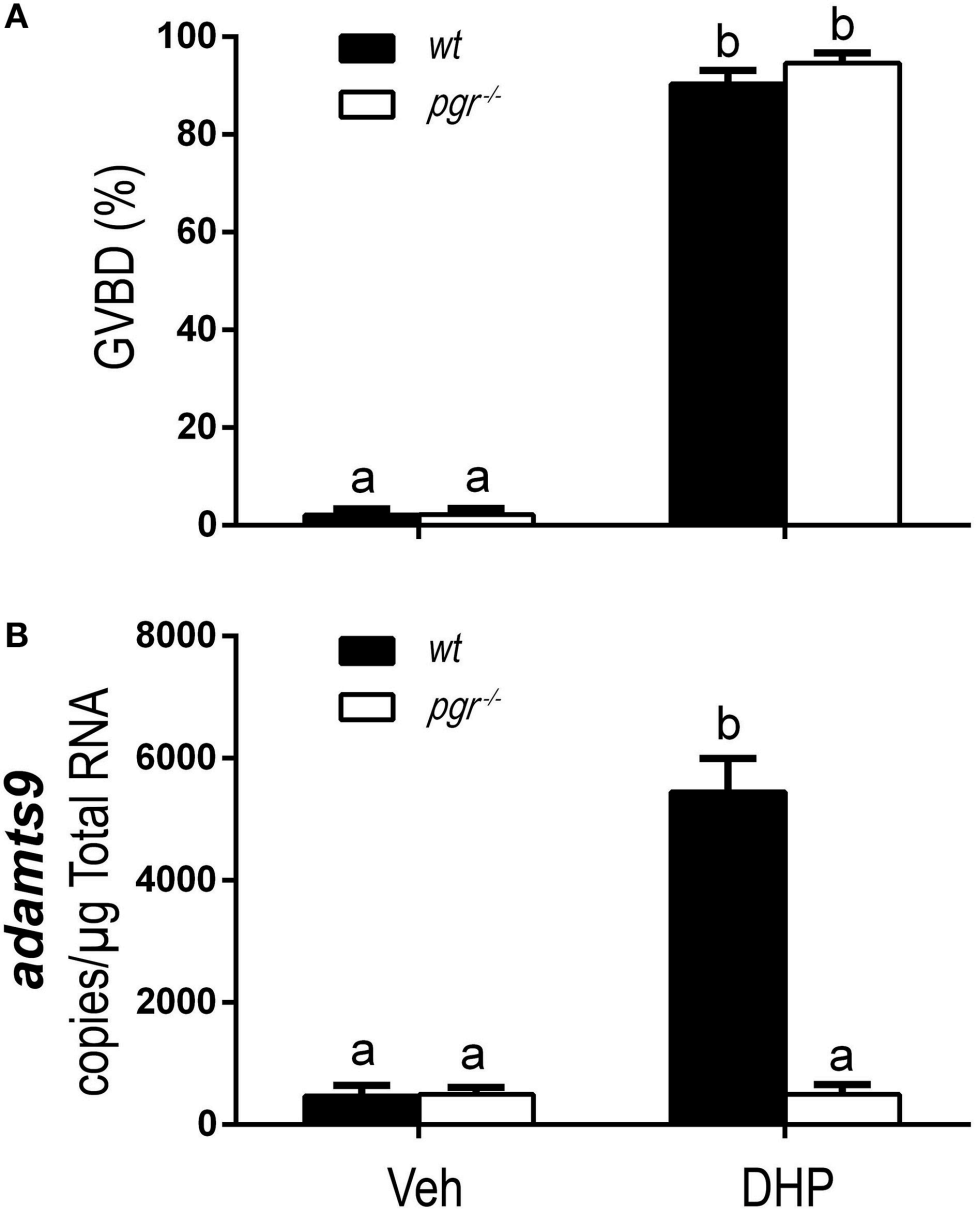
Complete inhibition of DHP induced *adamts9* expression in stage IVa follicles from Pgr knockout zebrafish in an *in vitro* incubation indicates a direct regulation of progestin in metalloproteinase. **(A)** Percentage of germinal vesicle breakdown (GVBD), **(B)** Expression of *adamts9* transcript. White bar, *pgr*^−/−^; black bar, *wt*. Different letters on the bars indicate significant differences (*p* < 0.05). Experiment was repeated three times with results of one representative experiment presented as mean ± SEM. *N* = 4.

As expected, DHP-induced oocyte maturation was not impaired in *pgr*^−/−^ fish (Figure 8A) because DHP signaling is mediated via the membrane progestin receptor (mPR) at the oocyte surface (24, 29, 31, 32). However, DHP-induced expression of *adamts9* was completely blocked in *pgr*^−/−^ fish *in vitro* (Figure 8B).

### DHP downregulates pgr protein

**Figure 9:**
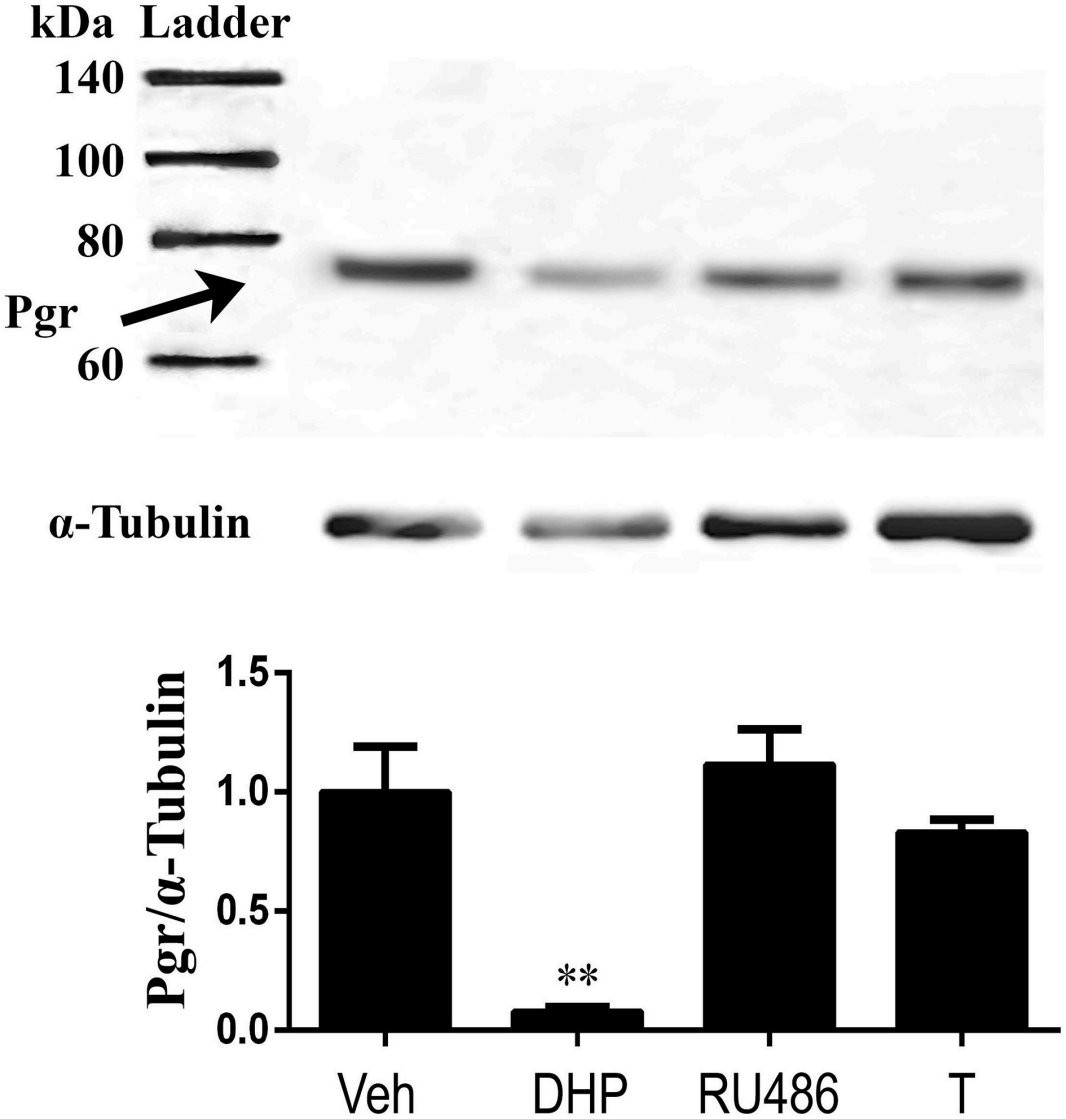
Exposure of exogenous DHP significantly reduced Pgr protein, while RU486 or T exposure had no effect on Pgr protein expression in stage IVa follicles in zebrafish *in vitro*. **(Top)** Representative images of a Western blot of Pgr proteins following treatments. **(Middle)** Representative image of a Western blot analysis of α-tubulin that was used as an internal control for normalization of expression of Pgr protein. **(Bottom)** Normalized Pgr expression in each treatment compared to vehicle control. Asterisks indicate significant difference compared to vehicle treatment. Experiment was repeated three times with results of one representative experiment presented as mean ± SEM. **p < 0.01.

Because we observed downregulation of Pgr during oocyte maturation (GVBD) *in vivo*, we hypothesized this downregulation of receptor is progestin (DHP) specific but not GVBD specific. Therefore, we tested our hypothesis by examining direct effect of DHP, RU486 and T on Pgr expression in an *in vitro* incubation of follicles. All three steroids induced GVBD in preovulatory follicles (stage IVa) following an 2.5 h *in vitro* incubation (data not shown). As expected, DHP treatment induced significant reduction of Pgr protein expression, whereas treatments of RU486 or T had no such effect on the expression of Pgr protein, comparable to the vehicle control (Figure 9).

## Discussion

In the present study, we provide the first evidence that expression of *adam8b, adamts1, adamts9*, and *mmp9* increased drastically within the preovulatory follicular cells of zebrafish, a basal vertebrate model. A significant increase of Pgr was required for this increase. Using follicles from *pgr*^−/−^ fish, we showed that expression of *adamts9, adam8b*, and *mmp9* was severely reduced in the knockout. One main cause for anovulation in *pgr*^−/−^ fish is likely due to this dramatically reduced expression of these metalloproteinases, which was caused by the loss of progestin signal in the Pgr knockout fish. Our study indicates a direct regulation of metalloproteinase by progestin and Pgr in preovulatory follicles, which likely is essential for ovulation in zebrafish.

In this study, we found Pgr was expressed throughout the daily ovulatory cycle in late stage follicles and was significantly elevated at the onset of oocyte maturation in zebrafish. This increase of Pgr corresponds to the transient appearance and increase of PGR prior to ovulation in mammals (2, 5, 29, 33–35), supporting the idea of conserved regulation and roles for Pgr in vertebrate ovulation (24, 26). Ligand-dependent down-regulation of PGR has been shown in multiple reproductive tissues in mammals (36, 37). This down-regulation of PGR was suggested to be concomitant with its transcriptional hyperactivity (38). In fact, production of DHP in zebrafish ovarian tissue increased significantly at 3.5 h prior to lights turned on (39), which could contribute to the down-regulation of Pgr in zebrafish. In the present study, Pgr protein level was downregulated either *in vitro* by DHP exposure or *in vivo* likely by endogenous progestin. Our results support previous studies and indicate that the appropriate Pgr expression in preovulatory follicles is required for the increase of metalloproteinase expression in zebrafish.

Suppression of the stimulatory effect of DHP on metalloproteinase expression in Pgr knockout indicates an indispensable role of Pgr in metalloproteinase expression in zebrafish ovary. Interestingly, expression of *adamts9* increased significantly during oocyte maturation *in vivo* in *pgr*^−/−^ fish, though the magnitude of increase was greatly reduced in *pgr*^−/−^ fish in comparison to those in *wt* fish. It has been suggested that LH and PGR have distinct but coordinate effects on transactivation of the *Adamts1* gene in mice granulosa cells (40). Whether gonadotropin stimulates *adamts9* and other metalloproteinase expression via Pgr-independent signaling pathway deserves further investigation. An interesting finding from the current study is failure of RU486 in inhibition of Pgr-dependent and DHP induced *adamts9* expression. One possible scenario is that RU486 has low or no binding affinity to zebrafish Pgr. A single amino acid substitution of glycine by cysteine in the hormone binding domain (HBD) of the human PGR abrogates binding of RU486 (41). Alignment of the zebrafish Pgr HBD with other species, including humans and chickens, has shown a glycine substitution by cysteine in the zebrafish Pgr (30, 42). Alternatively, instead of acting as an antagonist, RU486 can act as partial agonist (43). Indeed, our study shows that high doses of RU486 can induce oocyte maturation, which suggests that RU486 acts as an agonist of progestin activating mPRα and/or Pgr signaling in zebrafish. Further studies are required for elucidation of actions and molecular mechanisms of RU486 in fish.

Most likely, several proteinases are required for the appropriate remodeling of the extracellular matrix (ECM) and basal membranes that lead to follicular rupture and ovulation in vertebrates. Knockout of PGR, or administration of P4 inhibitors, significantly reduced hCG-induced expression of *Adam8* in mice granulosa cells (44). In agreement with this, we observed the expression of *adam8b* was also dramatically reduced in the follicular cells of *pgr*^−/−^ zebrafish, suggesting a conserved signaling pathway for the regulation of this metalloproteinase in preovulatory follicular cells. Unfortunately, knockout of Adam8 in mice had no noticeable effect on fertility, which might be due to the overlapping roles of other proteinases as multiple metalloproteinases could target the same substrate (15). MMPs have also been postulated to play critical roles in ECM remodeling associated with ovulation (45). Transcript of *MMP9* was elevated in rhesus monkey granulosa cells exposed to LH *in vitro* (46). Another study also suggested that MMP9 played a critical role in LH-induced steroidogenesis during ovulation in mouse granulosa cells (47). In line with these studies, we found significant increase of *mmp9* expression in zebrafish follicular cells prior to ovulation, and this increase was significantly reduced in *pgr*^−/−^ fish. Increase of *Adamts1* transcripts were observed in mice granulosa cells of periovulatory follicles after LH stimulus, but not in small follicles or in denuded oocytes (7). Other studies suggest that ADAMTS1 may cleave versican, a proteoglycan located in the basal follicular region and COC matrix (17). This cleavage of versican promotes changes in the ECM of preovulatory follicle required for the successful ovulation of a fertilizable oocyte in mice. In concert with these findings, our results showed that expression of *adamts1* increased remarkably in zebrafish follicular cells after oocyte maturation, supporting its role in ovulation. In human preovulatory granulosa cells, gene expression of *ADAMTS9* exhibited a significant upregulation (40-fold) following hCG treatment (48). In cattle, mifepristone inhibited the effects of LH on the expression of *ADAMTS9* transcripts in granulosa cells *in vitro* (8). In this study, we showed expression of *adamts9* in zebrafish was extremely low in immature follicles (stage I–IVa), negligible in denuded oocytes, and dramatically increased to its peak level specifically in the follicular cells of mature follicles (stage IVb) prior to ovulation. Additionally, knocking out Pgr indeed severely reduced the expression of *adamts9*. These results suggest that Adamts9 may also be responsible for follicular rupture. In summary, the preovulatory increases of *adam8b, mmp9, adamts1*, and *adamts9* in follicular cells support the requirement of multiple metalloproteinases for ovulation in zebrafish. Future studies including changes of protein contents and enzyme activities, and the effects of knockouts of these metalloproteinases in zebrafish are required to determine relative importance of each metalloproteinase in ovulation in zebrafish.

## Author contributions

DL performed experiments, analyzed results, and wrote the paper. NC, XW performed experiments. SC and WH supervised the project and discussed the results. YZ conceived and supervised the project, analyzed results, and wrote the paper.

### Conflict of interest statement

The authors declare that the research was conducted in the absence of any commercial or financial relationships that could be construed as a potential conflict of interest.

## References

[1] Kohda H Mori T Ezaki Y Nishimura T Kambegawa A. A progesterone-dependent step in ovulation induced by human chorionic gonadotrophin in immature rats primed with pregnant mare serum gonadotrophin. J Endocrinol. (1980) 87:105–7. 7430911 10.1677/joe.0.0870105

[2] Park OK Mayo KE. Transient expression of progesterone receptor messenger RNA in ovarian granulosa cells after the preovulatory luteinizing hormone surge. Mol Endocrinol. (1991) 5:967–78. 10.1210/mend-5-7-967 1840636

[3] Richards JS Russell DL Robker RL Dajee M Alliston TN. Molecular mechanisms of ovulation and luteinization. Mol Cell Endocrinol. (1998) 145:47–54. 10.1016/S0303-7207(98)00168-3 9922098

[4] Wlizla M Falco R Peshkin L Parlow AF Horb ME. Luteinizing Hormone is an effective replacement for hCG to induce ovulation in Xenopus. Dev Biol. (2016) 426:442. 10.1016/j.ydbio.2016.05.028 27263125 PMC5135639

[5] Richards JS Ascoli M. Endocrine, paracrine, and autocrine signaling pathways that regulate ovulation. Trends Endocrinol Metab. (2018). 29:315–25. 10.1016/j.tem.2018.02.012 29602523

[6] Robker RL Akison LK Russell DL. Control of oocyte release by progesterone receptor-regulated gene expression. Nucl Recept Signal (2009) 7:e012. 10.1621/nrs.07012 20087433 PMC2807638

[7] Robker RL Russell DL Espey LL Lydon JP O'Malley BW Richards JS. Progesterone-regulated genes in the ovulation process: ADAMTS-1 and cathepsin L proteases. Proc Natl Acad Sci USA. (2000) 97:4689–94. 10.1073/pnas.080073497 10781075 PMC18294

[8] Fortune JE Willis EL Bridges PJ Yang CS. The periovulatory period in cattle: progesterone, prostaglandins, oxytocin and ADAMTS proteases. Anim Reprod. (2009) 6:60–71. 20390049 PMC2853051

[9] Ogiwara K Takahashi T. Involvement of the nuclear progestin receptor in LH-induced expression of membrane type 2-matrix metalloproteinase required for follicle rupture during ovulation in the medaka, *Oryzias latipes*. Mol Cell Endocrinol. (2017) 450:54–63. 10.1016/j.mce.2017.04.016 28416325

[10] Curry TE Jr Osteen KG. The matrix metalloproteinase system: changes, regulation, and impact throughout the ovarian and uterine reproductive cycle. Endocr Rev. (2003) 24:428–65. 10.1210/er.2002-0005 12920150

[11] Ogiwara K Hagiwara A Rajapakse S Takahashi T. The role of urokinase plasminogen activator and plasminogen activator inhibitor-1 in follicle rupture during ovulation in the teleost medaka. Biol Reprod. (2015) 92:10. 10.1095/biolreprod.114.121442 25411388

[12] Carmeliet P Kieckens L Schoonjans L Ream B van Nuffelen A Prendergast G. Plasminogen activator inhibitor-1 gene-deficient mice. I Generation by homologous recombination and characterization. J Clin Invest. (1993) 92:2746–55. 10.1172/JCI116892 8254028 PMC288473

[13] Peschon JJ Slack JL Reddy P Stocking KL Sunnarborg SW Lee DC. An essential role for ectodomain shedding in mammalian development. Science (1998) 282:1281–4. 10.1126/science.282.5392.1281 9812885

[14] Vu TH Werb Z. Matrix metalloproteinases: effectors of development and normal physiology. Genes Dev. (2000) 14:2123–33. 10.1101/gad.815400 10970876

[15] Kelly K Hutchinson G Nebenius-Oosthuizen D Smith AJ Bartsch JW Horiuchi K. Metalloprotease-disintegrin ADAM8: expression analysis and targeted deletion in mice. Dev Dyn. (2005) 232:221–31. 10.1002/dvdy.20221 15580619

[16] Enomoto H Nelson CM Somerville RP Mielke K Dixon LJ Powell K. Cooperation of two ADAMTS metalloproteases in closure of the mouse palate identifies a requirement for versican proteolysis in regulating palatal mesenchyme proliferation. Development (2010) 137:4029–38. 10.1242/dev.050591 21041365 PMC2976286

[17] Brown HM Dunning KR Robker RL Boerboom D Pritchard M Lane M. ADAMTS1 cleavage of versican mediates essential structural remodeling of the ovarian follicle and cumulus-oocyte matrix during ovulation in mice. Biol Reprod. (2010) 83:549–57. 10.1095/biolreprod.110.084434 20592310

[18] Lydon JP DeMayo FJ Funk CR Mani SK Hughes AR Montgomery CA Jr. Mice lacking progesterone receptor exhibit pleiotropic reproductive abnormalities. Genes Dev. (1995) 9:2266–78. 7557380 10.1101/gad.9.18.2266

[19] Shimada M Nishibori M Yamashita Y Ito J Mori T Richards JS. Down-regulated expression of A disintegrin and metalloproteinase with thrombospondin-likerepeate-1 by progesterone receptor antagonist is associated with impaired expansion of porcinecumulus-oocyte complexes. Endocrinology. (2004) 145:4603–14. 10.1210/en.2004-0542 15231699

[20] Lima MA da Silva SV Freitas VM. Progesterone acts via the progesterone receptor to induce adamts proteases in ovarian cancer cells. J Ovarian Res. (2016) 9:9. 10.1186/s13048-016-0219-x 26916548 PMC4766681

[21] Ge W. Intrafollicular paracrine communication in the zebrafish ovary: the state of the art of an emerging model for the study of vertebrate folliculogenesis. Mol Cell Endocrinol. (2005) 237:1–10. 10.1016/j.mce.2005.03.012 15921848

[22] Spence R Gerlach G Lawrence C Smith C. The behaviour and ecology of the zebrafish, *Danio rerio*. Biol Rev Camb Philos Soc. (2008) 83:13–34. 10.1111/j.1469-185X.2007.00030.x 18093234

[23] Clelland E Peng C. Endocrine/paracrine control of zebrafish ovarian development. Mol Cell Endocrinol. (2009) 312:42–52. 10.1016/j.mce.2009.04.009 19406202

[24] Zhu Y Liu D Shaner ZC Chen S Hong W Stellwag EJ. Nuclear progestin receptor (pgr) knockouts in zebrafish demonstrate role for pgr in ovulation but not in rapid non-genomic steroid mediated meiosis resumption. Front Endocrinol. (2015) 6:37. 10.3389/fendo.2015.00037 25852646 PMC4365747

[25] Kubota K Cui W Dhakal P Wolfe MW Rumi MA Vivian JL. Rethinking progesterone regulation of female reproductive cyclicity. Proc Natl Acad Sci USA. (2016) 113:4212–7. 10.1073/pnas.1601825113 27035990 PMC4839436

[26] Liu DT Brewer MS Chen S Hong W Zhu Y. Transcriptomic signatures for ovulation in vertebrates. Gen Comp Endocrinol. (2017) 247:74–86. 10.1016/j.ygcen.2017.01.019 28111234 PMC5410184

[27] Wang C Liu D Chen W Ge W Hong W Zhu Y. Progestin increases the expression of gonadotropins in pituitaries of male zebrafish. J Endocrinol. (2016) 230:143–56. 10.1530/joe-16-0073 27113852 PMC4938713

[28] Selman K Wallace RA Sarka A Qi X. Stages of oocyte development in the zebrafish, *Brachydanio rerio*. J Morphol. (1993) 218:203–24. 10.1002/jmor.1052180209 29865471

[29] Hanna RN Zhu Y. Controls of meiotic signaling by membrane or nuclear progestin receptor in zebrafish follicle-enclosed oocytes. Mol Cell Endocrinol. (2011) 337:80–8. 10.1016/j.mce.2011.02.004 21335056

[30] Hanna RN Daly SC Pang Y Anglade I Kah O Thomas P. Characterization and expression of the nuclear progestin receptor in zebrafish gonads and brain. Biol Reprod. (2010) 82:112–22. 10.1095/biolreprod.109.078527 19741205 PMC2802116

[31] Zhu Y Rice CD Pang Y Pace M Thomas P. Cloning, expression, and characterization of a membrane progestin receptor and evidence it is an intermediary in meiotic maturation of fish oocytes. Proc Natl Acad Sci USA. (2003) 100:2231–6. 10.1073/pnas.0336132100 12574519 PMC151323

[32] Thomas P Pang Y Dong J Groenen P Kelder J de Vlieg J. Steroid and G protein binding characteristics of the seatrout and human progestin membrane receptor alpha subtypes and their evolutionary origins. Endocrinology (2007) 148:705–18. 10.1210/en.2006-0974 17082257

[33] Hild-Petito S Stouffer RL Brenner RM. Immunocytochemical localization of estradiol and progesterone receptors in the monkey ovary throughout the menstrual cycle. Endocrinology (1988) 123:2896–905. 10.1210/endo-123-6-2896 3197647

[34] Press MF Greene GL. Localization of progesterone receptor with monoclonal antibodies to the human progestin receptor. Endocrinology (1988) 122:1165–75. 10.1210/endo-122-3-1165. 3342750

[35] Bayaa M Booth RA Sheng Y Liu XJ. The classical progesterone receptor mediates Xenopus oocyte maturation through a nongenomic mechanism. Proc Natl Acad Sci USA. (2000) 97:12607–12. 10.1073/pnas.220302597 11050156 PMC18811

[36] Wei LL Krett NL Francis MD Gordon DF Wood WM O'Malley BW. Multiple human progesterone receptor messenger ribonucleic acids and their autoregulation by progestin agonists and antagonists in breast cancer cells. Mol Endocrinol. (1988) 2:62–72. 10.1210/mend-2-1-62 3398843

[37] Savouret JF Bailly A Misrahi M Rauch C Redeuilh G Chauchereau A. Characterization of the hormone responsive element involved in the regulation of the progesterone receptor gene. Embo J. (1991) 10:1875–83. 2050123 10.1002/j.1460-2075.1991.tb07713.x PMC452862

[38] Shen T Horwitz KB Lange CA. Transcriptional hyperactivity of human progesterone receptors is coupled to their ligand-dependent down-regulation by mitogen-activated protein kinase-dependent phosphorylation of serine 294. Mol Cell Biol. (2001) 21:6122–31. 10.1128/MCB.21.18.6122-6131.2001 11509655 PMC87329

[39] Lister AL Van Der Kraak GJ. Regulation of prostaglandin synthesis in ovaries of sexually-mature zebrafish (*Danio rerio*). Mol Reprod Dev. (2009) 76:1064–75. 10.1002/mrd.21072 19551897

[40] Doyle KM Russell DL Sriraman V Richards JS. Coordinate transcription of the ADAMTS-1 gene by luteinizing hormone and progesterone receptor. Mol Endocrinol. (2004) 18:2463–78. 10.1210/me.2003-0380 15256533

[41] Benhamou B Garcia T Lerouge T Vergezac A Gofflo D Bigogne C. A single amino acid that determines the sensitivity of progesterone receptors to RU486. Science (1992) 255:206–9. 1372753 10.1126/science.1372753

[42] Moudgil VK Lombardo G Hurd C Eliezer N Agarwal MK. Evidence for separate binding sites for progesterone and RU486 in the chick oviduct. Biochim Biophys Acta (1986) 889:192–9. 10.1016/0167-4889(86)90104-7 3778947

[43] Bagowski CP Myers JW Ferrell JE Jr. The classical progesterone receptor associates with p42 MAPK and is involved in phosphatidylinositol 3-kinase signaling in Xenopus oocytes. J Biol Chem. (2001) 276:37708–14. 10.1074/jbc.M104582200 11479298

[44] Sriraman V Eichenlaub-Ritter U Bartsch JW Rittger A Mulders SM Richards JS. Regulated expression of ADAM8 (a disintegrin and metalloprotease domain 8) in the mouse ovary: evidence for a regulatory role of luteinizing hormone, progesterone receptor, and epidermal growth factor-like growth factors. Biol Reprod. (2008) 78:1038–48. 10.1095/biolreprod.107.066340 18287572

[45] Cooke RG III Nothnick WB Komar C Burns P Curry JTE. Collagenase and gelatinase messenger ribonucleic acid expression and activity during follicular development in the rat ovary. Biol Reprod. (1999) 61:1309–16. 10.1095/biolreprod61.5.1309 10529279

[46] Duffy DM Stouffer RL. Luteinizing hormone acts directly at granulosa cells to stimulate periovulatory processes: modulation of luteinizing hormone effects by prostaglandins. Endocrine (2003) 22:249–56. 10.1385/ENDO:22:3:249 14709798

[47] Light A Hammes SR LH-induced steroidogenesis in the mouse ovary, but not testis, requires matrix metalloproteinase 2- and 9-mediated cleavage of upregulated EGF receptor ligands. Biol Reprod. (2015) 93:65 10.1095/biolreprod.115.130971 26203177 PMC4710187

[48] Rosewell KL Al-Alem L Zakerkish F McCord L Akin JW Chaffin CL. Induction of proteinases in the human preovulatory follicle of the menstrual cycle by human chorionic gonadotropin. Fertil Steril. (2015) 103:826–33. 10.1016/j.fertnstert.2014.11.017 25516084 PMC4346449

